# Uncertainty and Sensitivity Assessments of GPS and GIS Integrated Applications for Transportation

**DOI:** 10.3390/s140202683

**Published:** 2014-02-10

**Authors:** Sungchul Hong, Alan P. Vonderohe

**Affiliations:** 1 Korea Institute of Construction Technology, 283 Goyangdae-ro, Ilsanseo-gu, Goyang-si, Gyeonggi-do 411-712, Korea; 2 Vonderohe Consulting, LLC, W10751 Wildwood Way, Poynette, WI 53955, USA; E-Mail: vonderohe@centurytel.net

**Keywords:** Geographic Information System (GIS) for transportation, Global Positioning System (GPS), uncertainty analysis, sensitivity analysis

## Abstract

Uncertainty and sensitivity analysis methods are introduced, concerning the quality of spatial data as well as that of output information from Global Positioning System (GPS) and Geographic Information System (GIS) integrated applications for transportation. In the methods, an error model and an error propagation method form a basis for formulating characterization and propagation of uncertainties. They are developed in two distinct approaches: analytical and simulation. Thus, an initial evaluation is performed to compare and examine uncertainty estimations from the analytical and simulation approaches. The evaluation results show that estimated ranges of output information from the analytical and simulation approaches are compatible, but the simulation approach rather than the analytical approach is preferred for uncertainty and sensitivity analyses, due to its flexibility and capability to realize positional errors in both input data. Therefore, in a case study, uncertainty and sensitivity analyses based upon the simulation approach is conducted on a winter maintenance application. The sensitivity analysis is used to determine optimum input data qualities, and the uncertainty analysis is then applied to estimate overall qualities of output information from the application. The analysis results show that output information from the non-distance-based computation model is not sensitive to positional uncertainties in input data. However, for the distance-based computational model, output information has a different magnitude of uncertainties, depending on position uncertainties in input data.

## 1. Introduction

As the Geographic Information System (GIS) has been used for a wide range of transportation applications, positional errors inherent in spatial data become critical for ensuring spatial problem-solving and decision-making. However, GIS involves spatial data from multiple sources and different types. People are used to making decisions without knowledge of either positional errors in the data or their impact on output information. In GIS for transportation, various data-collection methods or devices have been used to maintain and update a spatial database, of which the Global Positioning System (GPS) provides a cost effective and efficient means of collecting spatial and non-spatial data along roadways. One emerging GPS-based method is to equip vehicles with Differential Global Positioning System (DGPS) receivers and numerous sensors [1–3]. All data coming from the vehicles are spatially and temporally referenced, and therefore they are adaptable in GIS.

However, positional uncertainties inevitably exist in GPS data points and roadway centerline maps. Although numerous map-matching algorithms have been proposed to correctly integrate GPS data points with a roadway centerline map [4–7], positional uncertainties still exist in snapped GPS-derived coordinates along roadway centerlines. These uncertainties increase and propagate to output products from GIS. Therefore, to make informed decisions, it is necessary to know the quality of output information associated with different levels of input data quality. Specifically, GIS applications should support optimum use of input data and, conversely, the optimum input for data use [8]. Considering the problems above, the following issues should be addressed when transportation agencies utilize GIS and GPS integrated applications:

- Characterization and propagation of positional uncertainties are not well formulated to determine a positional accuracy requirement for input data and a quality requirement for output information.
- Relative importance of multiple input data for output information is not well assessed and addressed.
- Overall uncertainties in output information are not well assessed and addressed.

Numerous approaches have been proposed to deal with problems concerning the quality of input data as well as that of output information from GIS applications such as hydrology, environment, and soil science [9–12]. However, GIS applications strongly depend on object type and data source [13]. In GPS and GIS integrated applications for transportation, input data are mostly GPS data points and a roadway spatial database, in which vehicles' trajectories are mostly represented with two-dimensional point features along with a one-dimensional roadway centerline. Thus, analytical- and simulation- based approaches were developed for modeling positional uncertainties in integrating GPS data points and GIS for transportation [14,15]. The primary driving factor in this study is the need for obtaining accurate and reliable information from the applications. Uncertainty and sensitivity analysis methods are, therefore, developed based upon the error modeling approaches. However, as they have different approaches of formulating characterization and propagation of positional uncertainties, it is essential to compare and evaluate those approaches before implementation to the applications. In this regard, the remainder of this paper is structured as follows: Section 2 conceptually illustrates the analytical- and simulation-based approaches for modeling positional errors and their propagation in the applications. Then, in Section 3, uncertainty estimations obtained by those approaches are compared and examined with test datasets, each of which has a different magnitude of complexity and curvilinearity. Section 4 presents the conceptual framework of the uncertainty and sensitivity analysis methods. In Section 5, for verification and demonstration purposes, the uncertainty and sensitivity analyses are conducted on a winter maintenance application to determine optimum input data as well as to estimate uncertainty properties of output information.

## 2. Error Modeling Approaches in Integrating GPS and GIS for Transportation

Modeling of positional errors and their propagation is necessary to understand error and its impact on GPS and GIS integrated applications for transportation. Generally, there are two approaches: analytical and simulation. The analytical approach estimates uncertainties in output information by applying the law of error propagation, assuming uncertainty properties of spatial data are known [16–18]. The simulation approach estimates positional errors by generating error-corrupted versions of the same spatial data. Displacements imposed on spatial data indicate a positional error distribution [19–21]. Monte Carlo simulation is used for error propagation. Since a sample generated from Monte Carlo simulation has statistical properties, an error impact on the applications can be analyzed with methods of statistical estimation and inference [22].

### 2.1. Analytical Approach

**Figure 1.**
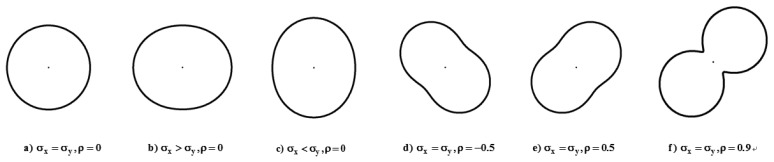
Analytical GPS error model (standard error curve).

The analytical approach involves the analytical GPS error model (or error curve) and the error propagation model, assuming that a test roadway centerline map is representative of roadway centerline maps with the same nominal scale [14]. Although an error ellipse is a typical error model for point measurements, it does not show the true shape of the standard error in all directions. Thus, an error curve is adopted as an analytical GPS error model [23]. The GPS error model has different shapes, sizes, and orientations depending on parameters in the covariance matrix (Figure 1).

Also, it implies a probability of proximity with other measurements of the same position when the same measurement technique is applied under the same conditions. The error model becomes circular when two variances are equal with no correlation. However, when there is high correlation, the error curve becomes highly curvilinear.

**Figure 2.**
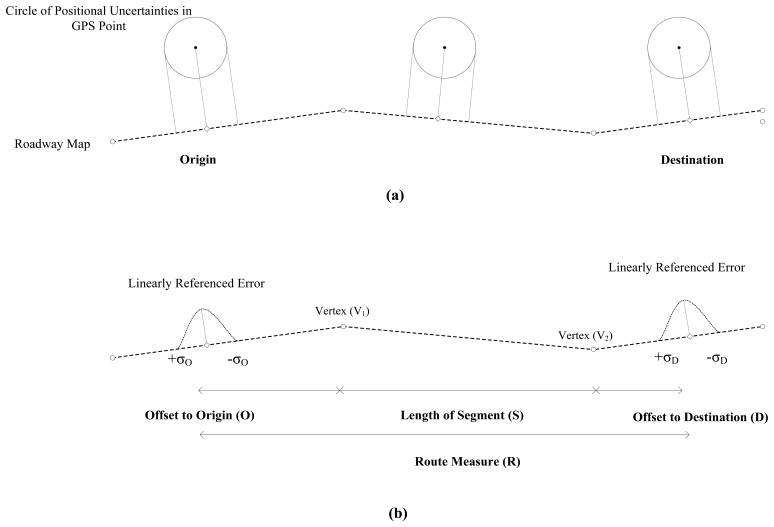
Uncertainty propagation in GPS and GIS applications for transportation. (**a**) GPS data points along roadway centerlines; and (**b**) Route measure computation.

When GPS points are integrated with a cartographic roadway network in GIS, the potential bounds of linearly-referenced errors are estimated by projecting the GPS error model onto roadway centerlines. For example, in Figure 2a, a vehicle equipped with a GPS receiver is represented as a series of point features. Roadway is depicted as roadway centerlines. Given that GPS errors along *x* and *y* axes are independent of each other, the GPS error model becomes a circle of positional uncertainties. GPS points are snapped to each roadway centerline with a linear transformation, including a snapping and a linear referencing. In Figure 2b, the route measure is determined with a segment length (*S*) and offsets to origin (*O*) and destination (*D*) (*R* = *O* + *S* + *D*). Also, the circles of uncertainties at origin and destination become linearly-referenced errors and propagate to a route measure. Uncertainties in the route measure, computed by the error propagation model [15], are then represented with $\sigma_{R}^{2}=\sigma_{O}^{2}+\sigma_{D}^{2}$. Also, when estimating uncertainties in output information that involves the computed route measure R as a parameter, the law of error propagation is repeatedly applied to computational models. For example, when output information is quantity of salt used for winter maintenance, a simple computational model is *Q* = *A* × *R*, where A is an average material application rate and R is a route measure. In this case, uncertainties in output information, estimated by the law of error propagation, are represented with $\sigma_{Q}^{2}=A^{2}\sigma_{R}^{2}$.

### 2.2. Simulation Approach

**Figure 3.**
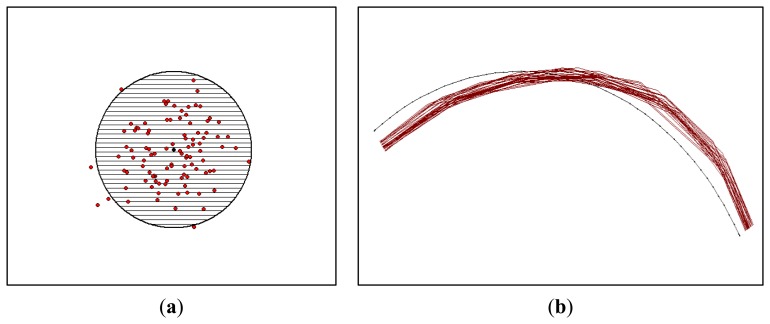
Simulation error model. (**a**) A GPS point; and (**b**) A roadway centerline map.

The simulation approach involves error models that generate a population of error-corrupted versions of GPS data points and roadway centerline map [15]. An error model for points has been well-studied for a long time in the areas of surveying, geodesy, and photogrammetry [24]. Point data can be perturbed according to an error ellipse prescribed by given parameters of a variance-covariance matrix. In Figure 3a, one hundred GPS data points are simulated, according to identical properties of the error ellipse with equal variances and zero covariance. As the error ellipse is shown with a 95% confidence region, the number of GPS points that fall within the error ellipse is close to the confidence region. However, for lines, the straightforward means to simply perturb their vertices reveals several limitations. A set of points comprising the line is closely related to a shape of linear feature and, consequently, is correlated. Thus, an error model was developed to simulate positional errors in roadway centerline maps. This simulation model estimates positional errors with respect to line segment lengths and vertex offsets, and generates error-corrupted versions of roadway centerline maps by introducing the estimated errors into a reference map (Figure 3b).

**Figure 4.**
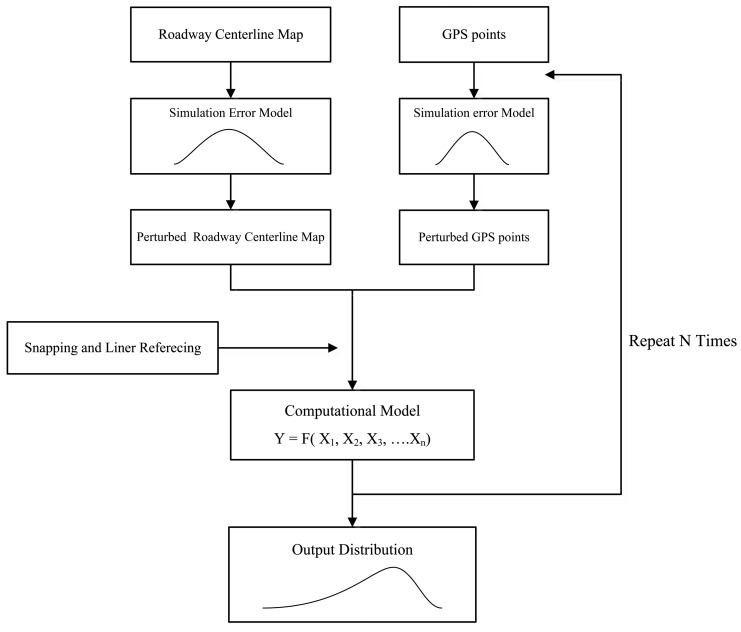
Simulation approach for modeling positional errors in GPS and GIS integrated applications for transportation.

The general procedure for the simulation approach is described in Figure 4. The first step for uncertainty analysis is to define the level of positional uncertainties in each input data. In the second step, the simulation error models will perturb input data to realize error-corrupted versions of GPS points and roadway centerline maps. At the same time, the snapping and linear referencing associate GPS points with a roadway centerline map. In the final step, output information is generated from a selected computational model. This procedure is repeated until a satisfactory distribution of the output information is obtained. In the approach, Monte Carlo simulation is used to translate uncertainties in input data to output information through spatial operations. A distribution of output information by the simulation reflects a quality of output information from given qualities of input data.

## 3. Evaluation of the Error Modeling Approaches: Analytical *versus* Simulation

### 3.1. Test Data and Areas

**Figure 5.**
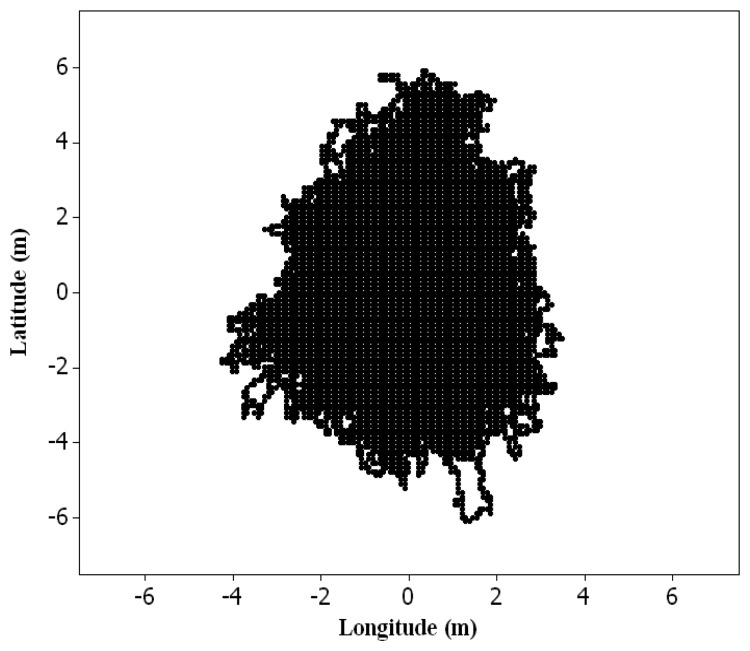
Positional uncertainties in DGPS data.

For comparison and evaluation of the analytical- and simulation-based approaches for modeling positional errors and their propagation in GPS and GIS integrated applications, the spatial data employed in this paper are DGPS data points from probe vehicles, and roadway centerline maps (Table 1). To model positional uncertainties in DGPS data, a stationary GPS data logger (Garmin Etrex Vista HCx) within a Wide Area Augmentation System (WAAS) mode was set up for 17 days (Figure 5), and its logging rate was 2 s, which is identical to the sampling rate of the DGPS datasets from probe vehicles.

**Figure 6.**
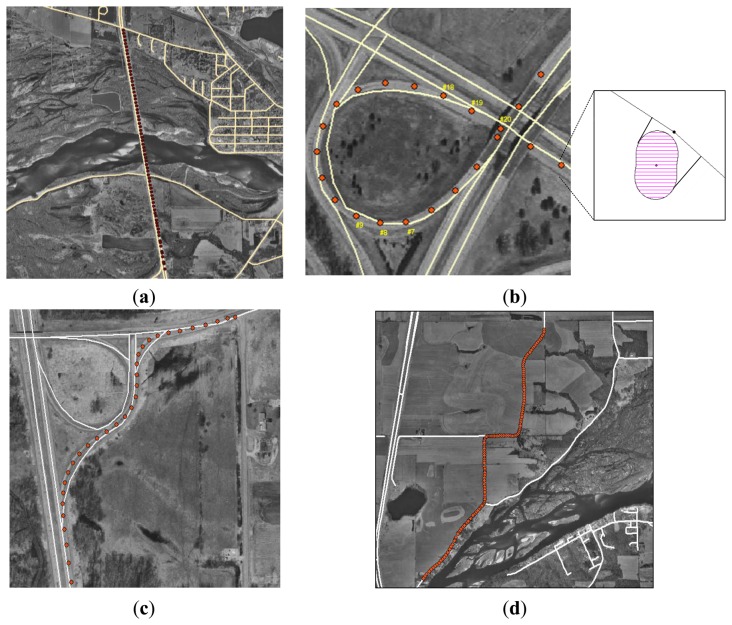
DGPS data points and roadway centerline maps on orthophotos. (**a**) Straightaway roadway; (**b**) Ramp 1; (**c**) Ramp 2; and (**d**) Curvilinear roadway.

Two roadway spatial databases with different nominal scales were obtained. The reference map, directly compiled from stereomodels by a mapping firm, depicts roadway centerlines with a 1:4,800 nominal scale. In an error modeling, the reference map is treated as representing the actual locations of roadways. The test map, obtained from a commercial vendor, was developed for vehicle navigation purposes. However, metadata for the test map were not available. The test map is assumed to have multiple sources, including maps of varying scales and DGPS-derived roadway centerline components from vehicles driven over roadways. Also, it is expected that different magnitudes of positional uncertainties exist depending upon the complexity and curvilinearity of roadways. Thus, test areas are divided into three groups: (1) straightaway roadway; (2) curvilinear roadway; and (3) ramp (Figure 6).

### 3.2. Application and Evaluation

For each test area shown in Figure 6, estimated ranges of route measures by the analytical approach are compared to that by the simulation approach. In Table 2, the quality of route measures estimated by those approaches is represented by a numerical value with a confidence interval. The numerical value is the best estimate of a route measure from given input data, and the confidence interval is a degree of uncertainty of the estimated route measure. Particularly, the analytical estimates of a route measure have symmetric or asymmetric confidence intervals, of which the asymmetric confidence intervals arise from DGPS data points at the beginnings and endings of a route being near vertices of the test map (Figure 6b). The simulation approach involves error models for both input data. When large uncertainties exist in roadway centerline maps such as ramps and curvilinear roadways, uncertainty estimations by the simulation approach tend to be larger than analytical estimates of uncertainty. But their estimates of a route measure are likely to be underestimated in comparison to analytical estimates, due to the correlated nature of positional errors realized by the simulation error model for roadway centerline maps.

For assessing the impact of positional uncertainties, a reference route measure from reference input data is compared to route measures estimated by the analytical and simulation approaches. However, different from the simulation approach, the analytical approach assumes that positional uncertainties in input data are independent. Their offsets and confidence intervals discretely reflect sensitivities to positional uncertainties in a roadway centerline map and DGPS data points, respectively. For example, in the straightaway roadway (Figure 6a), the analytical estimate of a route measure, which is close to the reference measure, indicates that the test map has no error source significantly affecting route measures. The similar range of a route measure by the simulation approach indicates that DGPS data points rather than the test map have a greater impact on uncertainties in a route measure. However, for the ramps and curvilinear roadway, sensitivities of the route measures sharply increase as the curvilinearity of roadways increases. A ramp is the one of the most complex features in highways, and it is highly subject to various types of error source. In Figure 6b,c, DGPS data points and test maps on orthophotos imply that uncertainties in route measures are influenced by alignment accuracy and insufficient polyline resolution. A different degree of positional uncertainties consequently exists in route measures, depending on the curvilinearity and complexity of roadways. Although the first ramp has the least route measure, the offsets in the analytical and simulation approaches are greater than that in other roadways.

Also, fundamental properties of the analytical and simulation approaches are identified. The analytical approach requires a complex mathematical process to model positional uncertainties in input data and their propagation through spatial operations. However, when a computational model for outputs is nonlinear, an error propagation model is simplified by applying a first-order Taylor series. Further simplification can be made by assuming that errors in input data are independent of one another. Thus, uncertainty estimation by the analytical approach is oftentimes unrealistic. Also, due to its inflexibility and impracticality, the analytical approach is limited to specific applications, even if positional errors in input data and utilized spatial operations are known. Different from the analytical approach, the computational cost of the simulation approach is heavy due to Monte Carlo simulation. But, as the spatial correlation of positional errors can be realized, the simulation approach is suitable for many situations to predict uncertainties in output information. Also, it can be applied to various applications due to its flexibility and simplicity.

### 4. Uncertainty and Sensitivity Analysis Method

Uncertainty and sensitivity analysis methods are developed based on the error modeling approaches. However, as evaluated in the Section 3, uncertainty estimations by the analytical approach are oftentimes unrealistic due to the independence assumption among positional errors. Even though heavy computational time is required, the simulation approach is utilized for uncertainty and sensitivity analyses due to its capability to realize positional errors in each input data type.

**Figure 7.**
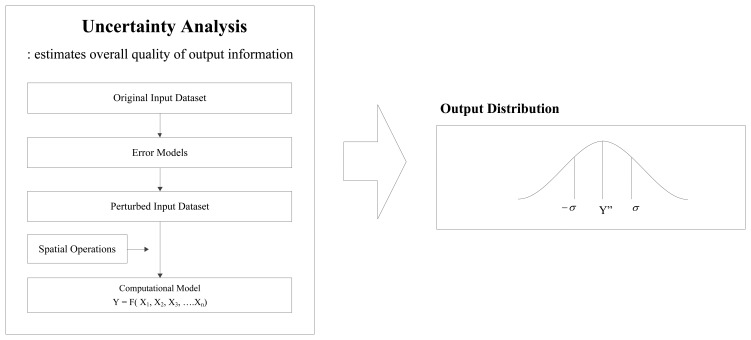
Conceptual view of uncertainty analysis method.

The uncertainty analysis method is designed to estimate the overall quality of output information based on spatial varieties of spatial data. A conceptual view of uncertainty analysis is illustrated in Figure 7, in which three functional components are an error model, a spatial operation, and a computation model. The error model is used to describe distributions of positional error in spatial data and to propagate the error through spatial processes so the quality of output information can be estimated. The spatial operation, referred to as a linear transformation, is used to associate GPS data points with a roadway centerline map. The computational model generates output information from the applications. A distribution of output information, finally obtained from the uncertainty analysis method, has two essential components: (1) a numerical value and (2) a degree of uncertainty. The numerical value is the best estimate of output information from given qualities of input data, and the degree of uncertainty is a quantitative indication of the reliability of the result.

**Figure 8.**
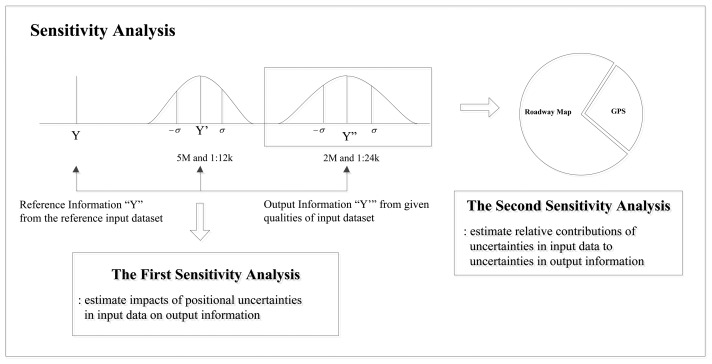
Conceptual view of sensitivity analysis.

In the development of GIS applications, data collection is the most important and expensive component. Thus, to determine optimal input data qualities for applications, a sensitivity analysis is designed to explore contributions of positional uncertainties in input data to variations in output information. The conceptual procedure for sensitivity analysis is illustrated in Figure 8.

The first sensitivity analysis is designed to estimate the impact of input data on output information with varying combinations of input data quality (e.g., 5 m accuracy GPS data and 1:12,000 scale roadway map; 2 m accuracy GPS data and 1:24,000 scale roadway map). Reference information from reference input data is compared to estimates of output information. The offset between estimated and reference information indicates impacts of positional uncertainties in input data on output information. On the other hand, the second sensitivity analysis is designed to estimate relative contributions of positional uncertainties in input data to uncertainties in output information. In this analysis, the confidence interval for output information is a degree of uncertainty that indicates a consistency (or repeatability) of output information from a given set of input data qualities. To measure the relative sensitivity, positional errors in one input data type are simulated while another input data type remains unperturbed.

### 5. Case Study: Winter Maintenance Application

#### 5.1. Overview of Uncertainty and Sensitivity Assessments

**Figure 9.**
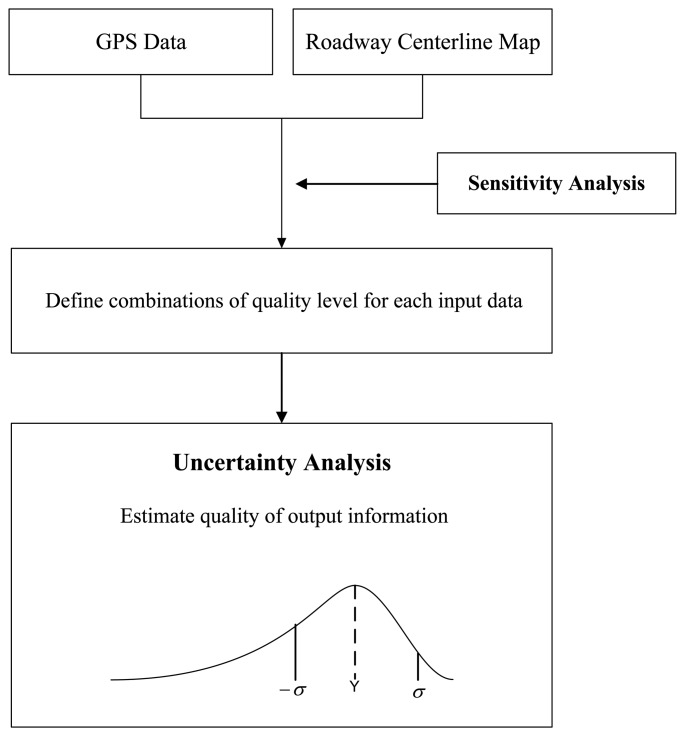
Overall procedure of uncertainty and sensitivity analyses.

The sensitivity and uncertainty analysis methods, proposed in this paper, provide means to formulate characterization and propagation of positional uncertainties in input data so the impact of the uncertainties on applications can be analyzed. For verification and demonstration purposes, the methods are applied to a winter highway maintenance application. The overall procedure for uncertainty and sensitivity analyses is illustrated in Figure 9. The first step is to characterize sensitivity of output information to positional uncertainties in input data, selected considering degrees of complexity and curvilinearity of roadways. In the applications, output information, computed from a non-distance-based computational model that only involves DGPS data points snapped to roadway centerlines, is not sensitive to positional uncertainties. Thus, sensitivity analysis is conducted for output information from a distance-based computational model that involves DGPS points and route measures. As a result, combinations of quality levels for each input data type are determined to obtain optimum quality in output information. The second step is to apply the uncertainty analysis method to describe overall qualities of output information from the applications. An output from the method is a distribution of output information, from which a numerical value with a confidence interval is derived.

#### 5.2. Winter Maintenance Application

**Figure 10.**
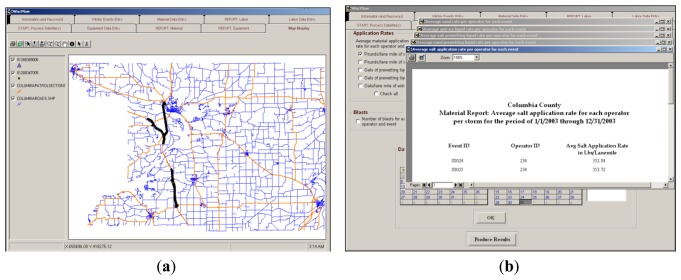
Winter Maintenance Application (WiscPlow). (**a**) Vehicles routes on map display; and (**b**) Performance measure.

**Figure 11.**
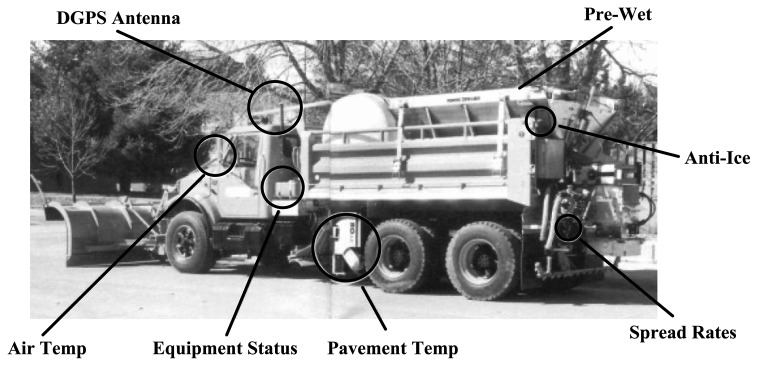
Winter maintenance vehicle wired with sensors and DGPS receiver.

The winter maintenance application, referred to as WiscPlow, was developed as a GPS and GIS application for winter storm reporting and resource management [1] (Figure 10). The datasets employed in this application are those collected by winter maintenance vehicles equipped with DGPS receivers and numerous sensors that collect speed, environmental data (e.g., pavement and air temperature), equipment status (e.g., plow up and plow down), and material usage (e.g., salt application rate) (Figure 11).

All data coming from the vehicles are represented as two-dimensional DGPS point features along roadway centerlines and have a temporal resolution equal to 2 s. WiscPlow calculates winter maintenance performance measures and develops analytical decision tools using spatial and non-spatial data. The performance measures, presented as reports and charts, provide a basis for decision-making on allocation of resources and enhancement of overall performance of winter operations [18]. In the application, computational models to generate performance measures are categorized as (1) a distance-based computational model and (2) a non-distance-based computational model. The distance-based computational model uses route measures and DGPS points snapped to roadway centerlines for computing output information, while the non-distance-based computational model uses only DGPS points snapped to roadway centerline.

#### 5.3. Computational Model

Computational models dealing with average salt application rate and total quantity of salt are selected to generate performance measures. The performance measure “Average salt application rate for each operator and event” in WiscPlow is computed by a non-distance-based computational model (the unit for the application rate is pounds of salt per lane mile):

(1) $${\text{MAR}}_{\text{salt},o,e}=[\sum_{y=1}^{Y_{\text{salt},o,e}}{\text{MAR}}_{\text{salt},y,o,e}]/2Y_{\text{salt},o,e}$$

where:

*MAR_salt_*_,_*_o_*_,_*_e_* : Average salt application rate for each operator and event
*MAR_salt_*_,_*_y_*_,_*_o_*_,_*_e_* : yth salt application rate reading during event and operator
*Y_salt_*_,_*_o_*_,_*_e_*: Total number of salt application rate readings during event and operator
*y* : Index for salt application rate reading.

The performance measure “Quantity of salt used for all events and each patrol section” is computed by a distance-based computational model (the unit for the quantity of salt is kilograms):

(2) $$Q_{\text{salt},p}=\sum_{e=1}^{E}Q_{\text{salt},p,e}$$

(3) $$Q_{\text{salt},p,e}=[\sum_{y=1}^{Y_{\text{salt},p,e}}MAR_{\text{salt},y,p,e}/2Y_{\text{salt},p,e}]\times L_{\text{salt},p,e}$$

where:

*Q_salt_*_,_*_p_* : Quantity of salt used for all events and each patrol section
*Q_salt_*_,_*_p,e_* : Quantity of salt used for each event and patrol section
*MAR_salt_*_,_*_y_*_,_*_p_*_,_*_e_* : yth salt application rate reading for patrol section and for the event
*Y_salt_*_,_*_p_*_,_*_e_*: Total number of salt application rate readings for event and patrol section
*L_salt_*_,_*_p_*_,_*_e_*: Number of treated lane miles in patrol section over which salt was distributed during event
*y* : Index for salt application rate reading, *E* : Total number of events, *e* : Index for event.

#### 5.4. Spatial Data Description

**Figure 12.**
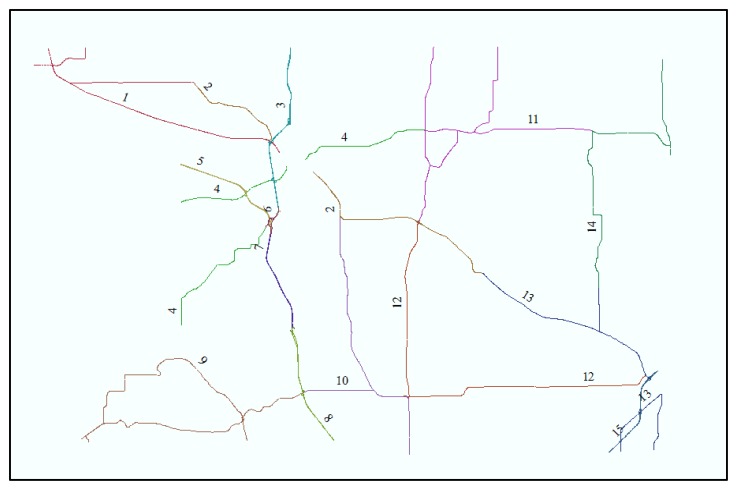
Patrol section map in Columbia County, WI, USA.

Spatial data covers a county roadway and an interstate highway in Columbia County, WI, USA (Figure 12). The coordinate system is the Columbia County Coordinate System based on the North American Datum 83 (NAD 83) and the measurement units are U.S. survey feet. Reference and test roadway spatial databases depict roadway centerlines with different nominal scales. The reference map, used in the previous section, is employed for modeling and simulating positional errors in the commercial and public maps shown in Table 3. The commercial map has 5 m accuracy in almost all areas. However, for the public map, roadway centerlines are available in limited areas and represented with a 1:24,000 nominal scale. The commercial map represents a limited resource but shows accurate roadway centerlines, while the public map represents an open source but coarsely depicts roadway centerlines. In the application, the functional class, attribute, and topology in the roadway spatial database are utilized for calculating performance measures with DGPS data from winter maintenance vehicles. DGPS data were collected over the 2001–2002 and 2002–2003 winter seasons, and it is assumed that the DGPS datasets have identical properties of positional uncertainty shown in Table 1.

#### 5.5. Sensitivity Analysis

**Figure 13.**
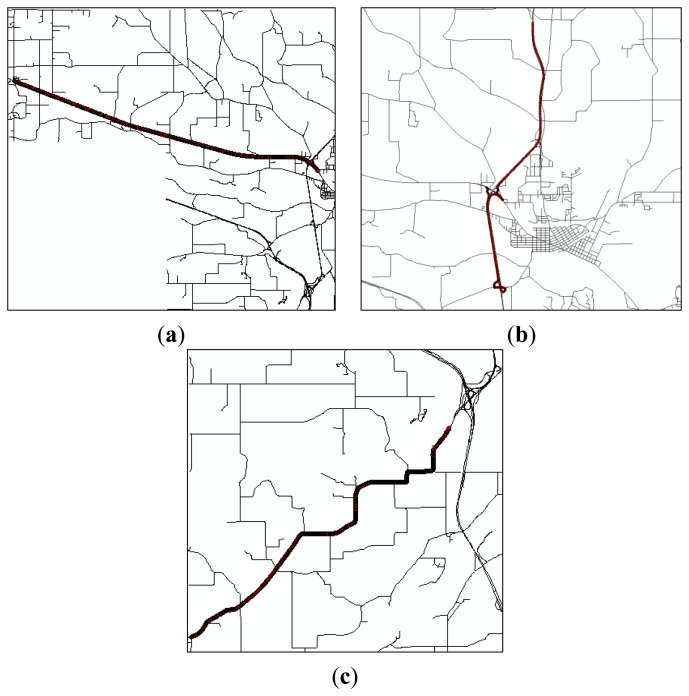
DGPS data points in patrol sections. (**a**) Patrol Section 1; (**b**) Patrol Section 3; and (**c**) Patrol Section 4.

The sensitivity analysis aims to characterize sensitivity of output information to positional uncertainties in input data, considering complexity and curvilinearity of roadways. The sensitivity analysis method was applied to roadway centerline maps and DGPS data points in Patrol Section 1, Patrol Section 3, and Patrol Section 4, which represent the straightaway roadway, the roadway including ramps, and the curvilinear roadway, respectively (Figure 13).

The first sensitivity analysis is designed to estimate the impact of errors in the roadway centerline map on performance measures, for which performance measures are computed from DGPS data points and roadway centerline maps altering from the commercial map to the public map. The reference performance measure from the reference input data is the basis for estimating the sensitivity of performance measures from test maps. However, “Average salt application rate for each operator and event,” computed from the non-distance-based computational model, is not sensitive to positional uncertainties in input data. Thus, the sensitivity analysis is conducted for “Quantity of salt used for all events and each patrol section” from the distance-based computational model.

Table 4 shows estimated ranges of “Quantity of salt used for all events and each patrol section.” For Patrol Section 1, the first sensitivity analysis shows that the commercial and public maps have a negligible impact on the performance measure. Regardless of nominal scales of the roadway centerline maps, estimated ranges of the performance measure have close estimates to the reference performance measure and small standard deviations. However, in Patrol Section 3 and Patrol Section 4, the sensitivities of performance measures to the roadway centerline maps sharply increase as the curvilinearity of roadways increases. In Patrol Section 3, the performance measure simulated from DGPS data points and the commercial map has a closer estimate to the reference performance measure than that from DGPS data points and the public map. Like the evaluation results, ramps are subject to various error sources. In addition to the alignment error and polyline resolution, uncertainties in the performance measure are influenced by different positions of entrances and exits of the ramp in the roadway centerline maps. Therefore, although the commercial map accurately depicts roadway centerlines, large degrees of uncertainty exist in the performance measure. For Patrol Section 4, the public map has a higher impact on the performance measure than the commercial map. Positional errors along curved roadway centerlines are accumulatively propagated into the performance measures as winter maintenance vehicles drive along the curvilinear roadways. As a result, the estimate of performance measure, which was simulated with the DGPS data and public map, deviates more from the reference measure, and its standard deviation is much greater than that simulated with the DGPS data points and commercial map.

For further analysis, the second sensitivity analysis method simulates route measures by the following combinations of error models: DGPS Data, roadway map, and DGPS Data and roadway map. Effects of input data on route measures are examined independently and in combination (Table 5). For Patrol Section 1, the sensitivity analysis shows that roadway centerline maps have a negligible impact on the performance measures. The uncertainty estimations by the DGPS error model are almost identical to the uncertainty estimations by error models of both input data. Thus, in the straight roadways, uncertainties in the performance measure are largely influenced by DGPS data points rather than by the roadway centerline map. For Patrol Section 3, the performance measure has high sensitivity to each input data type. But the roadway centerline map has a slightly larger impact on uncertainties in the performance measure comparing to DGPS data points. However, in Patrol Section 4, the degree of uncertainty in the roadway centerline map is relative to its nominal scale. The public map has a higher impact on uncertainties in the performance measure than do DGPS data points, but conversely, the commercial has a lower impact than do DGPS data points. Similar to other patrol sections, the summation of degree of uncertainty by each error model is larger than the degree of uncertainty from both error models. Therefore, the impact of errors in the input data is reduced while positional uncertainties are propagating through the spatial operations and computational model.

#### 5.6. Uncertainty Analysis

**Figure 14.**
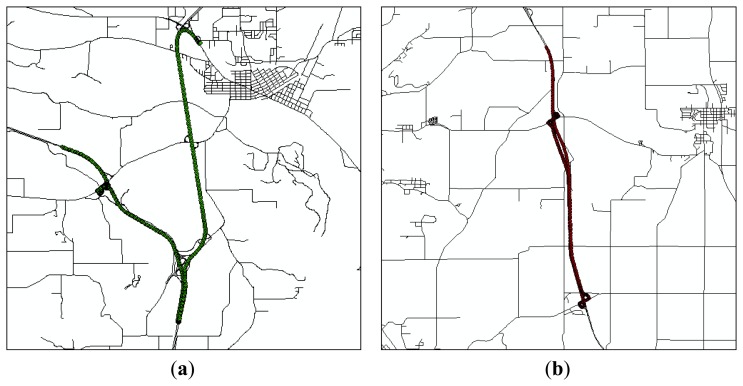
DGPS data points in Columbia County, WI, USA. (**a**) Operator ID: S0024; and (**b**) Operator ID: S0025.

When a combination of quality levels for each input data type is determined, uncertainty analysis was conducted on two datasets from winter maintenance vehicles driven by different operators in Figure 14. Tables 6 and 7 show overall uncertainties in performance measures. While an average salt application rate is calculated using snapped DGPS points, the quantity of salt is calculated by using both snapped DGPS points and route measures. Thus, the computational model for a quantity of salt is more sensitive than the computational model for average salt application rate. Results from uncertainty analysis also show that the average salt application rate for each event has zero uncertainty, but the quantity of salt used for each patrol section has a different degree of uncertainty depending on the treated lane miles and the curvilinearity of roadways. Moreover, uncertainties in quantity of salt will accumulate as the winter maintenance vehicles repeatedly treat patrol sections. With the above considerations, the commercial map rather than the public map is preferred for the winter maintenance application. Accurately defined roadway centerlines in the commercial map enable WiscPlow to compute more reliable performance measures.

### 6. Summary and Conclusions

The quality of spatial data becomes one of critical factors to be considered before utilizing GPS and GIS applications for spatial problem-solving and decision-making. For this reason, this paper introduced the sensitivity and uncertainty analysis methods that provide means for formulating characterization and propagation of uncertainties in the applications. The proposed methods analyze two aspects of uncertainty propagation: sensitivity analysis aims to estimate contributions of positional uncertainties in input data to variations in output information, and uncertainty analysis aims to predict an overall quality of output information from given qualities of input data.

In the uncertainty and sensitivity analyses, an error model and an error propagation method are basis for estimating the quality of output information from given qualities of input data. They are constructed in two distinct ways: an analytical approach and a simulation approach. Therefore, before implementation to the real application, the evaluation is preceded to compare and examine the analytical and simulation approaches. The evaluation results show that estimated ranges of output information from the analytical and simulation approaches are compatible. However, as the independence assumption among positional errors, uncertainty estimations by the analytical approach are oftentimes unrealistic. Even though heavy computational time is required, the simulation approach is more realistic due to error models for both types of input data. Therefore, in the development of the uncertainty and sensitivity analysis methods, the simulation approach rather than the analytical approach is preferred when considering the simplicity and flexibility for implementation.

For verification and demonstration purposes, the sensitivity and uncertainty analyses were conducted on the winter highway maintenance application. The sensitivity analysis method was used to determine input data qualities for the application. The uncertainty analysis method was then applied to estimate overall qualities of output information from the application. The results from the sensitivity and uncertainty analyses showed that consistent output information was calculated from the non-distance-based computation model, regardless of input data qualities. However, for the distance-based computational model, output information had a different magnitude of sensitivities depending on the input data quality and the spatial dependency in position errors along roadway centerlines.

In this paper, the uncertainty and sensitivity analysis methods were built upon positional error models for GPS data points and roadway centerline maps. However, GIS-T involves spatial data from various data collection devices and methods. Further research is necessary to develop error models for spatial measurements that are commonly applied to LRS (Linear Referencing System) such as distance measuring instruments, inertial measuring units, and dead reckoning systems. Also, the uncertainty and sensitivity analyses should be conducted under various environments affecting positional uncertainties in GPS data points and cartographic roadway network models. In addition, uncertainties in output information were most probable within acceptable bounds for decision-making, no matter the scale or quality of the available digital roadway centerline maps. Therefore, a rigorous approach needs to be designed to determine optimal levels of input data quality that produce acceptable levels of uncertainties in output information and, ultimately, to determine optimal quality of output information for decision-making with acceptable levels of risk.

## Figures and Tables

**Table 1.** Uncertainty properties in DGPS data.

**GPS Mode**	***X* Variance**	***Y* Variance**	**Correlation**
**DGPS**	1.46 m^2^	3.84 m^2^	0.06
**Map Type**	**Source**		**Nominal Scale**	
**Reference Map** **Test Map**	Columbia County, Wisconsin Land Information Office Commercial Vendor		1:4800 Unknown	

**Table 2.** Uncertainties in route measures *.

**Test Area**	**Reference Measure**	**Analytical Approach (m)**	**Simulation Approach (m)**
Straightaway Roadway	2456.56	2456.53 ± 2.74 (−)0.03	2456.62 ± 2.72 (0.06)
Ramp 1	660.38	$647.16_{-2.62}^{+2.51}\;(-13.22)$	645.86 ± 4.94 (−14.52)
Ramp 2	704.27	694.68 ± 2.46 (−9.59)	694.40 ± 2.66 (−9.87)
Curvilinear Roadway	3358.83	3368.24 ± 2.66 (9.41)	3365.64 ± 3.13 (6.81)

* Values in parentheses are offsets between estimated and reference route measures.

**Table 3.** Test maps in Columbia County, WI, USA.

**Test Map**	**Source**	**Nominal Scale (or Accuracy)**
**Commercial Map**	Commercial Vendor	5 m but 15 m in certain areas
**Public Map**	USGS 7.5- min quadrangles	1:24,000

**Table 4.** The first sensitivity analysis: “Quantity of salt used for all event and each patrol section (kg).”

**Item**	**Reference Input**	**DGPS & Commercial Map**	**DGPS & Public Map**
**Patrol Section 1**	999.75	999.71 ± 0.11	999.96 ± 0.12
**Patrol Section 3**	877.64	878.10 ± 0.69	876.30 ± 0.73
**Patrol Section 4**	693.59	693.38 ± 0.24	696.03 ± 0.56

**Table 5.** The second sensitivity analysis: “Quantity of salt used for all events and each patrol section (kg).”

**Simulation Model**			

**Item**	**DGPS**	**Commercial Map**	**DGPS & Commercial Map**
**Patrol Section 1**	999.74 ± 0.11	999.71 ± 0.03	999.71 ± 0.11
**Patrol Section 3**	878.15 ± 0.47	878.10 ± 0.54	878.10 ± 0.69
**Patrol Section 4**	693.64 ± 0.23	693.38 ± 0.05	693.38 ± 0.24

**Item**	**DGPS**	**Public Map**	**DGPS & Public Map**

**Patrol Section 1**	999.96 ± 0.11	999.96 ± 0.04	999.96 ± 0.12
**Patrol Section 3**	876.42 ± 0.48	876.30 ± 0.55	876.30 ± 0.73
**Patrol Section 4**	695.37 ± 0.24	696.03 ± 0.50	696.03 ± 0.56

**Table 6.** Uncertainty analysis: “Average salt application rate.”

**Operator ID**	**Average Salt Application Rate for Each Operator and Event (lbs./lane-mile)**
**S0024**	402.58 ± 0.00
**S0025**	17.09 ± 0.00

**Table 7.** “Uncertainty analysis: Quantify of salt.”

**Item**	**Quantify of Salt Used for** **All Events and Each Patrol Section (kg)**	**Treated Lane-Miles** **(lane-mile)**
**Patrol Section 3**	113.33 ± 0.38	3.46
**Patrol Section 4**	14.49 ± 0.36	0.13
**Patrol Section 5**	739.10 ± 0.99	5.14
**Patrol Section 6**	80.36 ± 0.23	0.60
**Patrol Section 7**	211.20 ± 0.80	2.43
**Patrol Section 8**	8.57 ± 0.43	0.42
